# Interferometric Imaging, and Beam-Formed Study of a Moving Type-IV Radio Burst with LOFAR

**DOI:** 10.1007/s11207-022-02042-0

**Published:** 2022-09-09

**Authors:** Hongyu Liu, Pietro Zucca, Kyung-Suk Cho, Anshu Kumari, Peijin Zhang, Jasmina Magdalenić, Rok-Soon Kim, Sujin Kim, Juhyung Kang

**Affiliations:** 1 Center for Magnetic Materials and Devices, College of Physics and Electronic Engineering, Qujing Normal University, Qujing, 655011 China; 2 Korea Astronomy and Space Science Institute, 776 Daedeokdae-ro, Yuseong-gu, Daejeon, 34055 Korea; 3 University of Science and Technology, 217 Gajeong-ro, Yuseong-gu, Daejeon, 34113 Korea; 4 ASTRON, the Netherlands Institute for Radio Astronomy, Oude Hoogeveensedijk 4, Dwingeloo, 7991PD the Netherlands; 5 Department of Physics, University of Helsinki, Pietari Kalmin katu 5, 00560 Helsinki, Finland; 6 Institute of Astronomy and National Astronomical Observatory, Bulgarian Academy of Sciences, Sofia, 1784 Bulgaria; 7 Solar-Terrestrial Center of Excellence–SIDC, Royal Observatory of Belgium, Av. Circulaire 3, 1180 Brussels, Belgium; 8 Center for Mathematical Plasma Astrophysics, Department of Mathematics, KU Leuven, Celestijnenlaan 200B, 3001 Leuven, Belgium; 9 Astronomy Program, Department of Physics and Astronomy, Seoul National University, Seoul, 08826 Republic of Korea

**Keywords:** Radio bursts, Type-IV bursts, Radio emission, Theory

## Abstract

Type-IV radio bursts have been studied for over 50 years. However, the specifics of the radio emission mechanisms is still an open question. In order to provide more information about the emission mechanisms, we studied a moving Type-IV radio burst with fine structures (spike group) by using the high-resolution capability of the Low-Frequency Array (LOFAR) on August 25, 2014. We present a comparison of Nançay Radioheliograph (NRH) and the first LOFAR imaging data of the Type-IV radio burst. The degree of circular polarization (DCP) is calculated at frequencies in the range 20 – 180 MHz using LOFAR data, and it was found that the value of DCP gradually increased during the event, with values of 20 – 30%. LOFAR interferometric data were combined with white-light observations in order to track the propagation of this Type-IV burst. The kinematics shows a westward motion of the radio sources, slower than the CME leading edge. The dynamic spectrum of LOFAR shows a large number of fine structures with durations of less than 1 s and high brightness temperatures ($T_{ \mathrm{B}}$), i.e., $10^{12}$ – $10^{13}$ K. The gradual increase of DCP supports gyrosynchrotron emission as the most plausible mechanism for the Type IV. However, coherent emissions such as Electron Cyclotron Maser (ECM) instability may be responsible for small-scale fine structures. Countless fine structures altogether were responsible for such high $T_{\mathrm{B}}$.

**Supplementary Information:**

The online version contains supplementary material available at 10.1007/s11207-022-02042-0.

## Introduction

The Sun is an active star, and its energetic phenomena is frequently accompanied by electromagnetic emissions over a wide spectral range including radio wavelengths. Solar radio bursts can show up in dynamic spectra, in which the features of increased intensity of the burst in comparison to the background emission can be identified. They are classified at meter wavelengths into five major types, Type I to Type V named after Roman numbers (Boischot, 1957). Solar radio bursts are generally related to coronal mass ejections (CMEs) and solar flares (Morosan et al., 2021). In this paper we focus on a Type-IV radio burst, which is a long-lasting broadband continuum emission in radio wavelengths that usually appears approximately 10 minutes after a solar flare (Pick, 1963). They are often considered to be generated by a process involving energetic particles trapped in a postflare loop (Stewart, 1985) and/or outward propagating magnetic structures (Bastian et al., 2001; Vasanth et al., 2016; Lv et al., 2021), such as the CME magnetic fields (Vasanth et al., 2019). Depending on whether the radio source propagates, we can distinguish a moving Type-IV radio burst (m-Type-IV) from a stationary Type-IV radio burst (Kundu, 1965; Pick, 1986). Due to its close relation with CMEs, the m-Type-IV has been frequently addressed in solar radio physics since its discovery.

The emission mechanism of Type-IV radio bursts is still highly controversial (see, e.g., Kuijpers, 1974; Morosan et al., 2019; Lv et al., 2021). Boischot (1957) first observed Type-IV radio emission and proposed synchrotron emission as the mechanism. However, since a high degree of circular polarization (DCP) (>20%) is observed during some Type-IV events, Kai (1969) suggested gyrosynchrotron emission instead. Benz and Tarnstrom (1976) concluded that Type-IV bursts of narrow bandwidth are generated by plasma emission instead of gyrosynchrotron emission. Later, Duncan (1981) considered plasma emission to explain the observed high brightness temperature ($T_{\mathrm{B}}$). In addition to the already mentioned emission mechanisms, Winglee and Dulk (1986) discussed the possibility of electron cyclotron maser (ECM) emission alongside the possibility of plasma emission (Upper Hybrid mode). Recent studies have featured gyrosynchrotron radiation (Carley et al., 2017), plasma emission (Vasanth et al., 2019), and ECM emission (Liu et al., 2018) as the possible emission mechanisms of the Type-IV continuum.

One important characteristic of the Type-IV continuum is its fine structures (Aurass et al., 1987; Chernov, 2011). Recent studies have reported fine structures of very short durations (4 to 60 ms at half-power of the burst) (Magdalenić et al., 2006; Chernov, 2008; Katoh et al., 2014). Fine structures may also have different appearances on radio dynamic spectra. The most frequently observed fine structures of the Type-IV continuum are zebra patterns (Tan, 2010; Kaneda et al., 2018), fiber bursts (Bernold and Treumann, 1983; Antonov et al., 2014), spikes (Benz, 1986), and slowly drifting narrowband structures (Katoh et al., 2014). Since fine structures usually have higher brightness temperature than background Type-IV emission, they are more likely to be generated by a different emission process.

Early studies of solar radio bursts were focused on understanding their basic physical characteristics employing single-frequency observations and dynamic spectra. With the development of radio imaging instruments, more studies have been conducted to track the source of radio bursts on the Sun. Since the Nancay Radioheliograph (NRH) started its observations in May 1956 (Blum, Boischot, and Ginat, 1957), metric-wavelength (i.e., <500 MHz) imaging study of the radio Sun has a history of more than 60 years. The Culgoora radioheliograph was the second one of the type, starting observations from February 1968 (Wild, 1970), but imaging observations stopped working in 1980s. In 1997, the Gauribidanur Radioheliograph (GRAPH; Ramesh et al., 1998) in India came into use, as an addition to the existing NRH instrument that can observe Stokes I and V. In January 2015, NRH imaging observations stopped, to undergo extensive maintenance. Observations were resumed in November 2020, and currently only Stokes I is observed, as well as the radio image of the Sun at ten separate frequencies within the range of 150 – 445 MHz (Kerdraon and Delouis, 1997). With the help of NRH, a number of studies have been carried out addressing Type-IV continuum emission (Bouratzis et al., 2016; Koval et al., 2016). The Low-Frequency Array (LOFAR) (van Haarlem et al., 2013) in the Netherlands is not a solar-dedicated instrument but during regular solar-dedicated campaigns, the Sun is observed in a similar frequency range as that by NRH. There have been several studies on solar Type-III (e.g., Morosan et al., 2014; Reid and Kontar, 2018; Zhang et al., 2020a), Type-II radio bursts (e.g., Chrysaphi et al., 2018; Zucca et al., 2018; Magdalenić et al., 2020; Maguire et al., 2021), and short-duration bursts (e.g., Morosan et al., 2015; Zhang et al., 2020b) using LOFAR data. Type-IV radio bursts on the other hand, have been reported less often with LOFAR observations. Gordovskyy et al. (2019) performed a statistical study of the positions of solar radio bursts, among which two were Type-IV events. The authors found a discrepancy between the obtained radio-source locations and the expected radial height as mapped by the Newkirk density model. This discrepancy was attributed to the strong wave scattering due to plasma turbulence in the active corona.

In our study, we examine a moving Type-IV radio burst with its fine structures observed by LOFAR on August 25, 2014. Despite its long history, the emission mechanism of Type-IV bursts is not fully understood. This study will contribute to a better understanding of Type-IV bursts based on the high-resolution observation of LOFAR. In Section 2, we show an overview of the Type-IV radio burst and associated solar activity. A comparison of NRH and LOFAR imaging data is then presented.

In Section 3, we calculate the degree of circular polarization of the Type-IV burst. In addition, we combine the LOFAR imaging data with SDO/AIA and Solar and Heliospheric Observatory/Large Angle and Spectrometric Coronagraph (SOHO/LASCO)-C2 to track the Type-IV radio sources and understand their association with the ambient coronal structures. Finally, we present details of fine structures and the estimated brightness temperature, followed by a discussion on the radiation mechanism. This is the first detailed analysis of a Type-IV radio burst with LOFAR interferometric imaging data.

## Observations and Data Analysis

### The Low-Frequency Array and Data Preparation

The Low-Frequency Array (LOFAR) utilizes omnidirectional antennas to form a phased array. LOFAR operates in the 10 MHz to 240 MHz frequency range with a frequency resolution of 12.5 kHz. There are two types of antennas: Low Band Antenna (LBA) and High Band Antenna (HBA), observing two different frequency ranges 10 – 90 MHz and 110 – 240 MHz, respectively. The frequency range is subject to change according to the LOFAR stations used to carry out the observation. While conventional radioheliographs like NRH can only provide Stokes I and V data, LOFAR simultaneously observes Stokes I, Q, U, and V (van Haarlem et al., 2013). As one of the newest radio telescopes to date, LOFAR greatly improved the angular resolutions of solar observations (Mann, Vocks, and Breitling, 2011).

**Figure 1:**
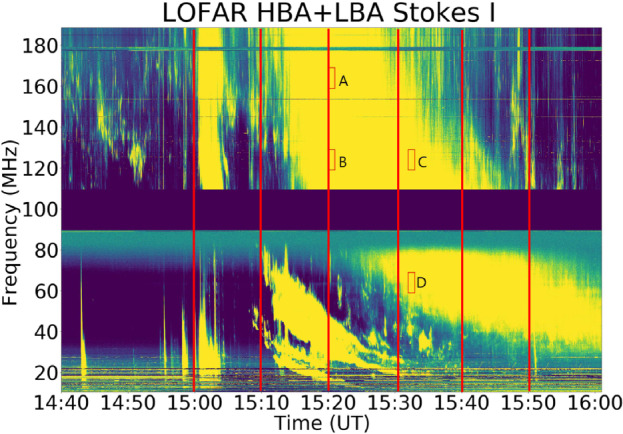
LOFAR radio dynamic spectrum on Aug 25, 2014 from 14:40 UT to 16:00 UT. Type-III radio bursts appeared in both HBA (110 – 190 MHz) and LBA (10 – 90 MHz) at 15:00 UT, then a Type-II radio burst appeared in LBA at 15:09 UT. At 15:16, a Type-IV radio burst appeared in HBA and later in LBA. A, B, C, and D are four selected areas within the Type-IV radio burst, and zoom-in plots of these areas are shown in Figure 7. The vertical lines on the spectrum in red color are the time stamps where the polarization profiles were estimated in Figure 4.

We carried out imaging spectroscopy observations of the Sun with LOFAR on August 25, 2014. For LOFAR observations of this event, the frequency range of LBA is 10 MHz to 90 MHz, and that of HBA is 110 MHz to 190 MHz. The dynamic spectrum in this work is obtained from a core station. The flux value in the dynamic spectrum is the uncalibrated relative flux intensity with the median background subtracted for each frequency channel, the combined dynamic spectrum of LBA and HBA is shown in Figure 1.

The interferometric imaging used the data from both LOFAR core and remote stations, imaging data is available only for HBA in this event. The measurement set is preprocessed with the Default Pre-Processing Pipeline (DPPP; van Diepen, Dijkema, and Offringa, 2018). The preprocessing includes three steps. First, we derive the amplitude and the phase solutions from the analysis of the observation of Virgo-A with a Virgo-A model. Secondly, we apply the solutions to the solar observations to perform the calibration. Finally, we apply the LOFAR-beam corrections to the calibrated visibility. After the preprocessing, we used the WS-Clean (Offringa et al., 2014; Offringa and Smirnov, 2017) for the Fourier transform and deconvolution to obtain the radio flux image. The calibration in SFU is a straightforward calculation inferred by knowing the beam size of LOFAR and by observing at the same time a calibrator. For more details see, e.g., de Vos, Gunst, and Nijboer (2009). The imaging and calibration process to obtain the physical unit (brightness temperature in [K]) uses the LOFAR-Sun toolkit with similar procedures as recent solar radio imaging works (Zhang et al., 2020a, 2022). The LOFAR channels at 125, 160, and 165 MHz suffer from calibration issues. In the observation of the calibrator for these frequencies, the long baseline is influenced by the solar radio emission or RFI from other sources, thus the crosscalibration can not be accomplished in these channels. The technical details of imaging can be found in the documentation in the code repository (https://github.com/peijin94/LOFAR-Sun-tools).

The coordinate system of the imaging result is transformed from Equinox with J2000 epoch to the helioprojective coordinate system. In the lower-right corner of the radio image of this day, there are some projected fake sources. Hence, we have also applied a fade-out fit to the calibrated data on the lower-right corner to erase the fake sources. This does not affect the actual radio sources of the event. All the processes mentioned above were done with the ASTRON CEP3 server, and visualization was done using the Solar and Space Weather KSP routines (https://git.astron.nl/ssw-ksp/lofar-sun-tools) using SunPy codes (SunPy Community et al., 2015).

### Event Overview

On Aug 25, 2014, an M2.0 class flare occurred in NOAA 12146, originating from the NOAA active region (AR) 12146, starting at 14:46 UT.^1^ Meanwhile, the SOHO/LASCO CME catalog provided by NASA Goddard Space Flight Center (Gopalswamy et al., 2009), reports a halo CME first seen in the SOHO/LASCO C2 field of view at 15:36 UT.^2^ The CME speed is reported to be 555 km s^−1^. The eruptive CME/flare event was associated with a complex Type-III/II/IV radio burst shown by LOFAR combined dynamic spectrum (HBA and LBA) in Figure 1.

In Figure 1, a group of Type-III radio bursts associated with the impulsive phase of the long-duration flare is visible in both HBA and LBA at 15:00 UT, followed by a Type-II radio burst in LBA at 15:09 UT (Magdalenić et al., 2020). At 15:10 UT, a Type-IV radio burst was observed, first in HBA and later in LBA. Despite the observation gap between 90 MHz and 110 MHz, it is clear that the Type-IV radio continuum spans from the HBA to the LBA range. The frequency drift of the continuum emission implies that this is a moving Type-IV radio burst. Another group of Type-III bursts was observed at about 15:48 UT in HBA. In this study, we focus only on the Type-IV emission.

### LOFAR Imaging Data of the Sun and Comparison with NRH

**Figure 2:**
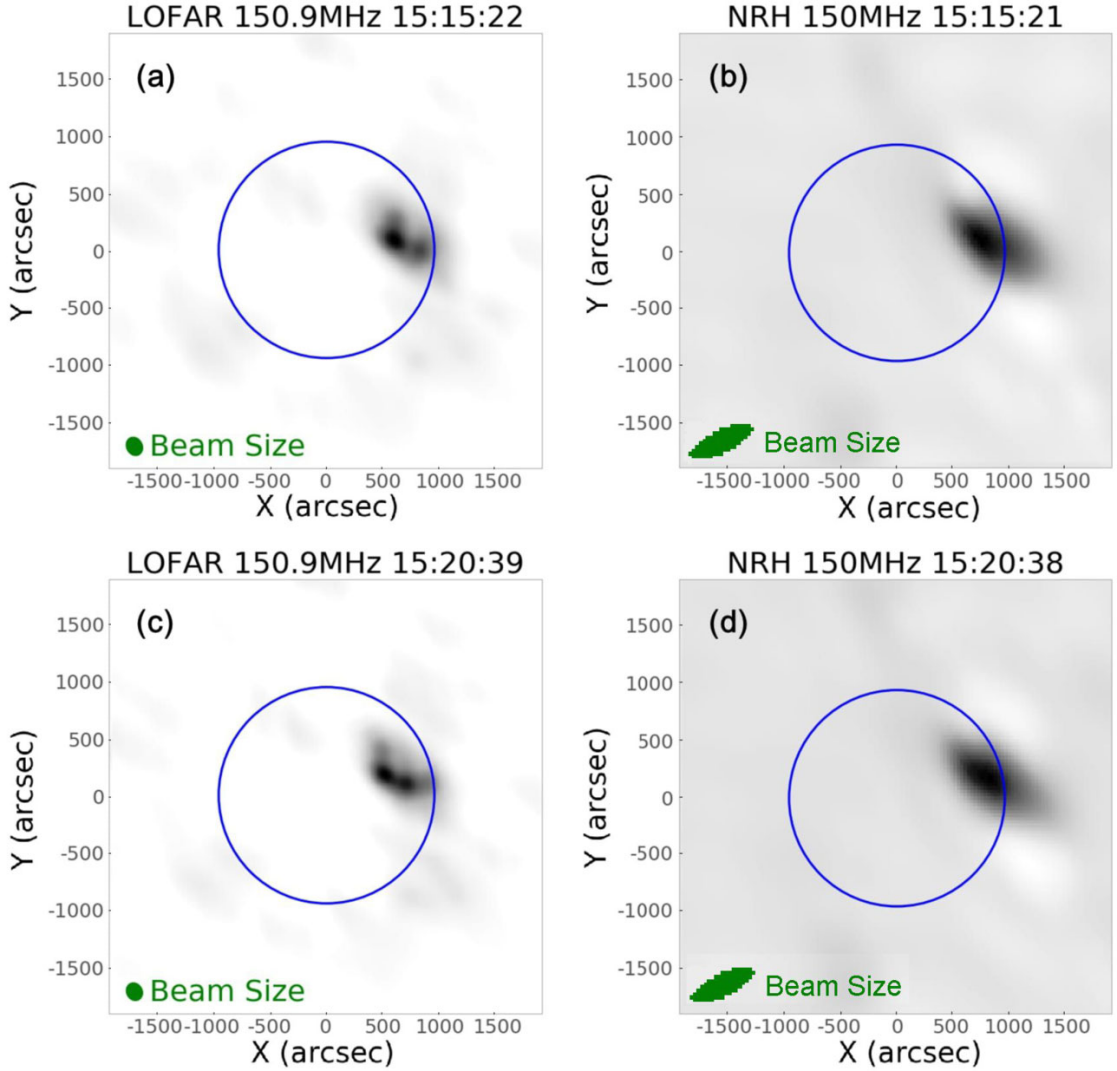
Comparison of LOFAR and NRH imaging observations of the Sun at 150.9 MHz on Aug 25, 2014. (**a**) and (**b**) are, respectively, LOFAR and NRH observations of the Sun at 15:15 UT while (**c**) and (**d**) are the same observations at 15:20 UT. The solar limb is indicated by a blue circle. Beam sizes of LOFAR and NRH are indicated by green ellipses in (**a**) and (**c**), and (**b**) and (**d**), respectively. All images represent the position of the Type-IV continuum source. (An animation of this figure is available online.)

We performed a detailed comparison of LOFAR and NRH imaging data. The comparison of NRH and LOFAR spatial resolution is important because we employ LOFAR observation with the remote baselines to demonstrate the advantages in beam size and resolution of a long baseline. For this event, LOFAR HBA has observations from 110 to 190 MHz. Two NRH frequency channels (150.9 MHz and 173 MHz), overlap with LOFAR observations. On Aug 25, 2014, LOFAR had data from 12:12 UT until 16:12 UT, while NRH observed the Sun until 15:23 UT. Since LOFAR has a frequency resolution of 12.5 kHz, radio imaging data can be produced to match the exact NRH frequency. After acquiring the cleaned LOFAR imaging data of the Sun, we rescaled LOFAR data to make the field of view identical to that of NRH, and plotted LOFAR 150 MHz and NRH 150 MHz observations together. A movie is available in the online material. Some of the frames are shown in Figure 2.

Both movie and images show that the positions of LOFAR and NRH radio sources are in agreement. In Figure 2(a), we can see multiple separate sources on LOFAR, while in Figure 2(b), we can only see one faint source on NRH. Similarly, in Figure 2(c), at least three separate structures are visible in LOFAR images, while in Figure 2(d), we can only see a blurry radio source. The beam size (∼ 200 arcsec along major axes) of LOFAR is shown on the lower-left corner in Figures 2(a) and (c), while the beam size (∼ 650 arcsec along major axes) of NRH is shown on the lower-left corner in Figures 2(b) and (d). Since the beam size of LOFAR is smaller than the displacement of the separate radio sources (∼ 500 arcsec), we clearly observe two sources that are separated and resolved. We are able to resolve the multiple sources at the onset of the Type-IV that appear as a single large source in NRH with a 1.8 km baseline. This indicates the importance of medium baselines 3 to 10 km to resolve complex radio sources and understand better the evolution and kinematics of the event.

## Results

### Degree of Circular Polarization

A solar radio burst has many physical parameters, and they are crucial not only to determine the emission mechanism, but also to understand the ambient coronal conditions (Melrose, 1975). One of the important parameters of the radio bursts is the degree of circular polarization (DCP). The degree of circular polarization of solar emission is given by (Collett, 1992):

1 $$\mathrm{DCP}=\frac{V}{I},$$

where V and I are the corresponding Stokes parameters. LOFAR provides full Stokes parameters, i.e., Stokes I, Q, U, and V. As often observed for radio instrumentation, a small amount of Stokes V in the LOFAR HBA band has leaked into Stokes U. In fact, the polarization leakage (linear spill of intensity from Stokes V to U) is a common instrumental issue in the majority of radio instruments (Kumari et al., 2021; Morosan et al., 2022). We simply correct the leakage by using the following process since LOFAR provides four Stokes parameters. In order to obtain the real Stokes V data in HBA, we introduced some corrections. Thus, the DCP of HBA is calculated as follows:

2 $$|{\mathrm{DCP}}_{\mathrm{corr}}|=\frac{\sqrt{V^{2}+U^{2}}}{I}.$$

**Figure 3:**
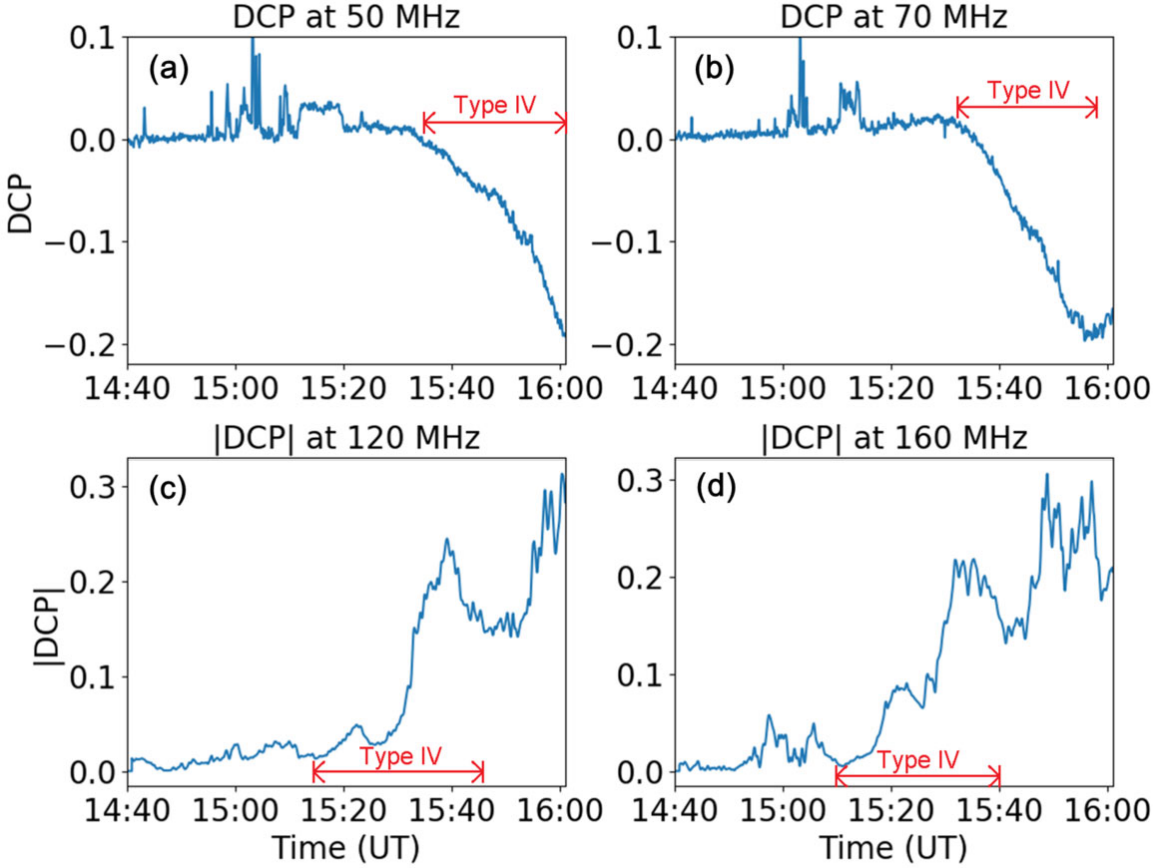
Degree of circular polarization at four frequencies. (**a**) and (**b**) are DCP at 50 MHz and 70 MHz, respectively; (**c**) and (**d**) are the absolute values of DCPs at 120 MHz and 160 MHz. The time range is the same as that of Figure 1. Time span of Type IV is shown on each panel. Note that the difference in the Type-IV span between (**a**), (**b**) and (**c**), (**d**) is due to the frequency drift of the burst.

The signal leakage to Stokes U is proportional to the Stokes V intensity, making it a clear instrumental effect. Moreover, this correction can be confidently carried out for the Sun as we can assume that the linear components of the incoming radio waves are removed by Faraday rotation (FR) in the corona. Figure 3 shows DCP diagrams from 14:40 UT to 16:00 UT at 50 MHz and 70 MHz calculated using Equation 1. The presented absolute value of DCP at 120 MHz and 160 MHz was estimated using Equation 2.

Figure 3 shows that the DCP of this Type-IV continuum amounts to 10 – 20%, and the DCP value rises as Type IV develops in time. The increasing DCP after the end of the Type-IV burst, as shown in Figures 3(c) and (d), is due to the presence of other types of radio bursts superposed at higher frequencies. The Type-I- and Type-III-like sources result in the increase of DCP visible. This is a good indicator that the instrument is capable of measuring DCP well. The increase at 15:40 UT at 120 MHz is a clear sign of the superposition of the Type-III and Type-I sources. We can still show well that the Type IV (especially where well isolated in the profiles at 50 and 70 MHz) shows a clear trend of decreasing DCP. This can be done thanks to the full Stokes observations of LOFAR. From Figures 3(a) and (b), we conclude that the DCP of this Type IV is negative, which means that left-hand circular polarization dominates. To preserve the sign of the DCP we use the following equation:

3 $${\mathrm{DCP}}_{\mathrm{corr}}=\frac{\left|V\right|}{V}\frac{\sqrt{V^{2}+U^{2}}}{I}.$$

We also plot the DCP along vertical lines on the spectrum in Figure 1. For this, we have selected six time instances from the spectra, which includes the DCP during no bursts observed and during the Type-II, -III, and -IV events. The DCP is calculated by averaging the 1 min spectral data and implementing the correction as shown in Equation 3.

**Figure 4:**
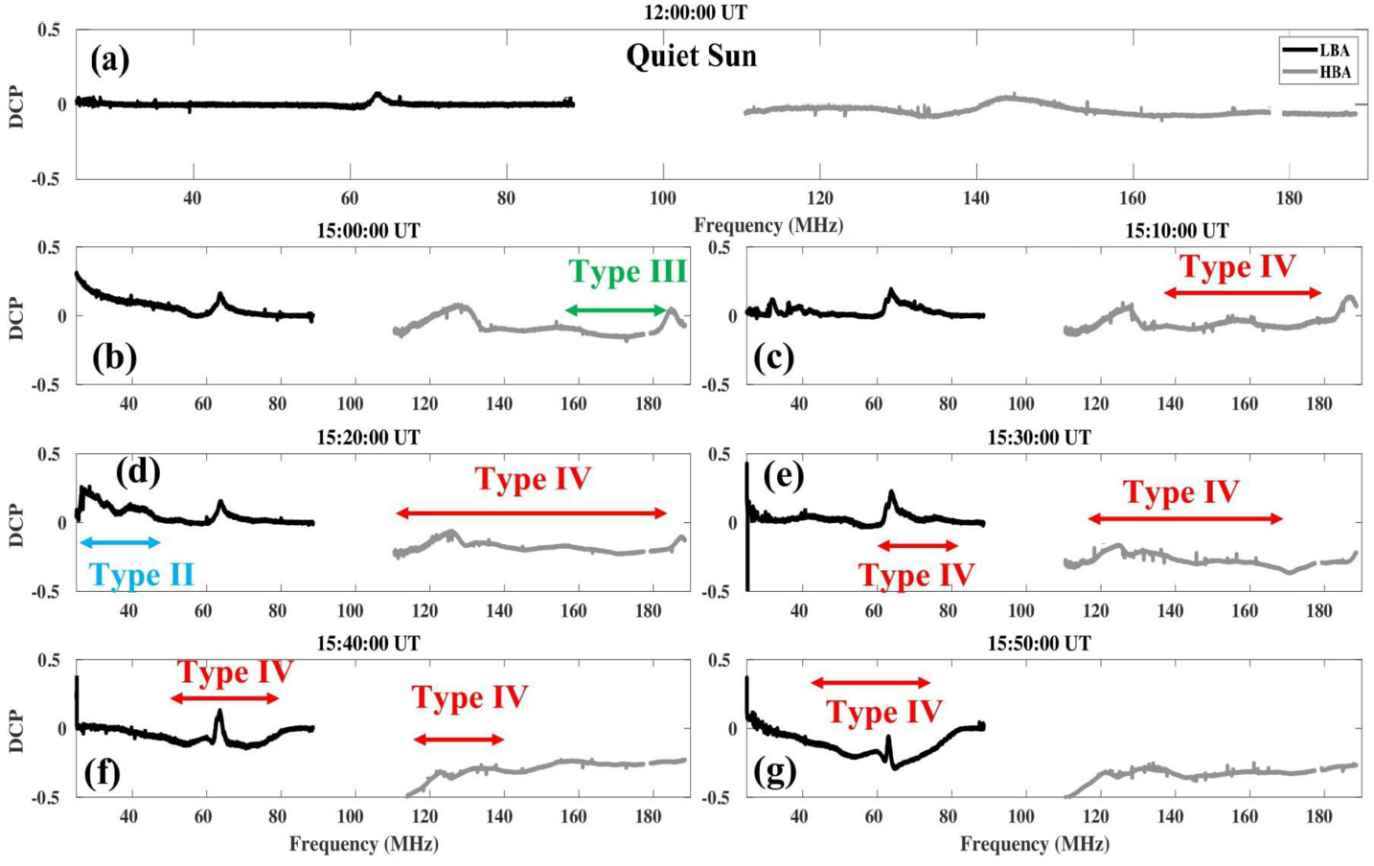
(**a**): DCP variation along the full LOFAR frequency range at 12:00 UT where no bursts are observed (quiet Sun); (**b**): Corrected DCP along the full LOFAR frequency range during Type-III radio bursts in HBA; (**c**) to (**g**): Corrected DCP along the full LOFAR frequency range during a Type-IV event, for five different instances; and (**d**): Corrected DCP during Type-II event in LBA.

Figure 4(a) shows the DCP variation along the full LOFAR frequency range at 12:00 UT when no bursts are present. The flat profile with very little modulation shows that in the absence of radio bursts, the instrument records almost no DCP. Panels (b) to (g) of Figure 4 show the DCPs (again along the full LOFAR frequency range) at six different times when there are radio bursts recorded. The time intervals during which different types of radio bursts were recorded are marked with arrows of different colors. Starting from about 15:00 UT a group of Type-III radio bursts is visible in the spectrum, while from 15:10 UT to 15:50 UT, the Type IV that we study is present (see also Figure 1). A gradual change of the DCP towards a negative value is observed as the Type-IV radio burst propagates away from the Sun (Figure 4 panels (d) to (g)). We note that at 63 MHz, there is a spike that can be seen at all considered times. This spike is due to radio-frequency interference (RFI).

Although the observed gradual rise of DCP value favors gyrosynchrotron radiation as the generation mechanism (Dulk, 1985), other physical parameters such as $T_{\mathrm{B}}$, also need to be considered in order to determine the emission mechanism of this Type-IV continuum.

### Propagation of Radio Sources

**Figure 5:**
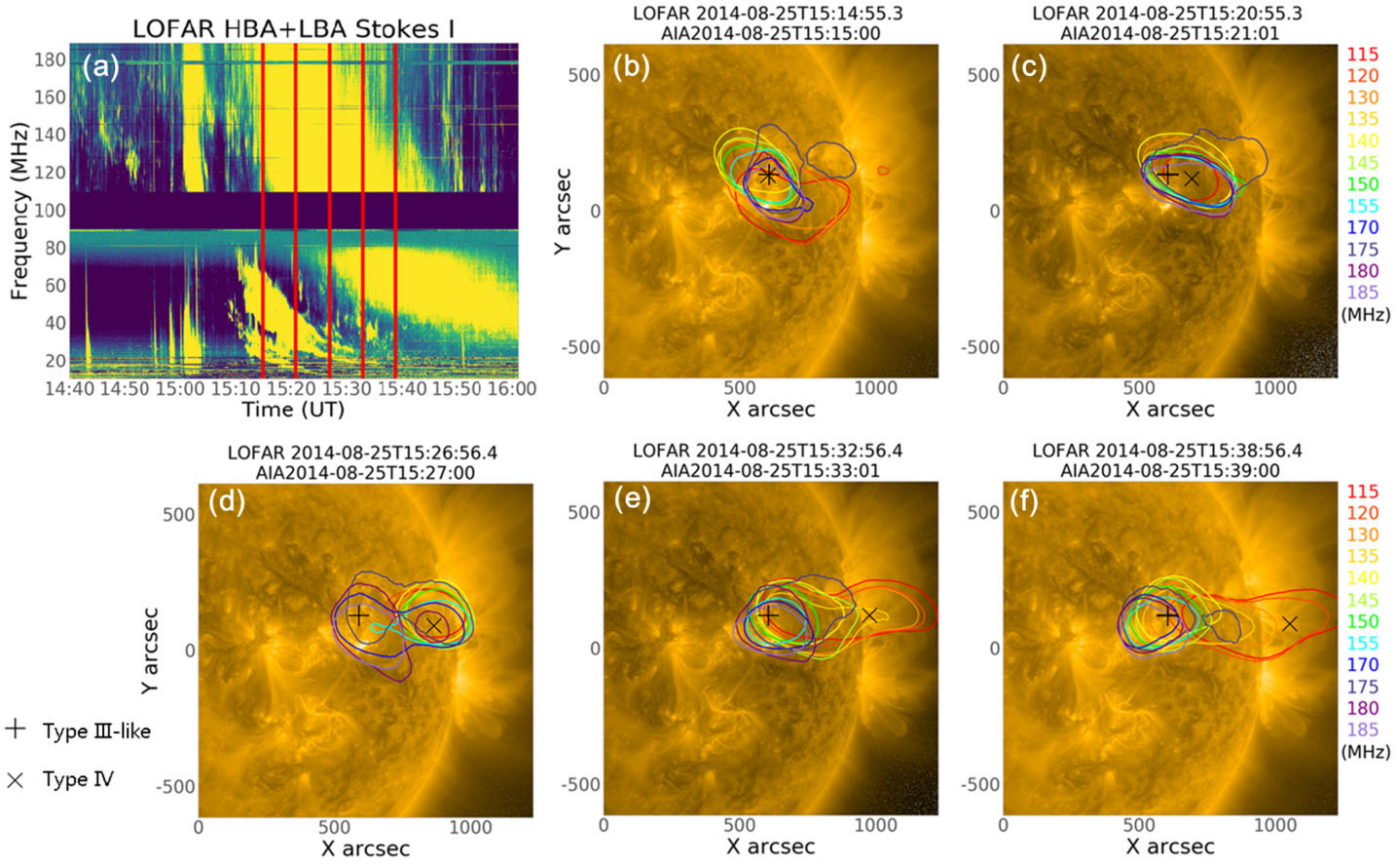
(**a**): LOFAR HBA and LBA dynamic spectrum on Aug 25, 2014. Five red vertical lines indicate the time of panels (**b**)–(**f**). In the panels (**b**)–(**f**): SDO/AIA 171 Å images are overplotted with LOFAR 70% maximum contours at every 5 MHz from 115 to 185 MHz, with the exception of 125, 160, and 165 MHz. (**b**): All LOFAR frequencies start to group near the active region; (**c**): Frequency contours propagate to the west limb of the Sun; (**d**): High-frequency (170 – 185 MHz) contours start to warp back to the active region, from where Type-III-like structures originated; (**e**) and (**f**): only three low frequency contours keep propagating westward while all remaining frequencies are again near the active region. (An animation of this figure is available online.)

In order to investigate the properties of the Type-IV continuum and its association with the ambient coronal structures, we plotted LOFAR 70% maximum contours of different frequencies in SDO/AIA 171 Å images. The movie is provided as online material and a few frames are shown in Figures 5(b)–(f).

At 15:15 UT, all radio sources appeared to be situated above the active region (NOAA AR 12146) that was the source of the associated CME/flare event. In Figure 5(c), almost all frequencies seem to propagate toward the west limb of the Sun. At 15:27 UT, radio sources at higher frequencies (170 – 185 MHz) started to be observed again closer to the active region. This behavior of the radio sources is expected because the continuum emission decreases in intensity and is no longer observed at higher LOFAR frequencies (see Figure 1). We therefore start to observe again the radio sources associated with the active region. At the same time, Type-IV emission at lower frequencies of the HBA band can still be observed until about 15:48 UT. In Figures 5(e) and (f), only three low-frequency (115 – 130 MHz) contours kept propagating westward, while all remaining frequencies map the faint radio source, associated with the active region. An expanding postflare loop was not clearly seen in AIA 171 Å running difference images. This might be due to the low emissivity of such loops in EUV wavelengths.

**Figure 6:**
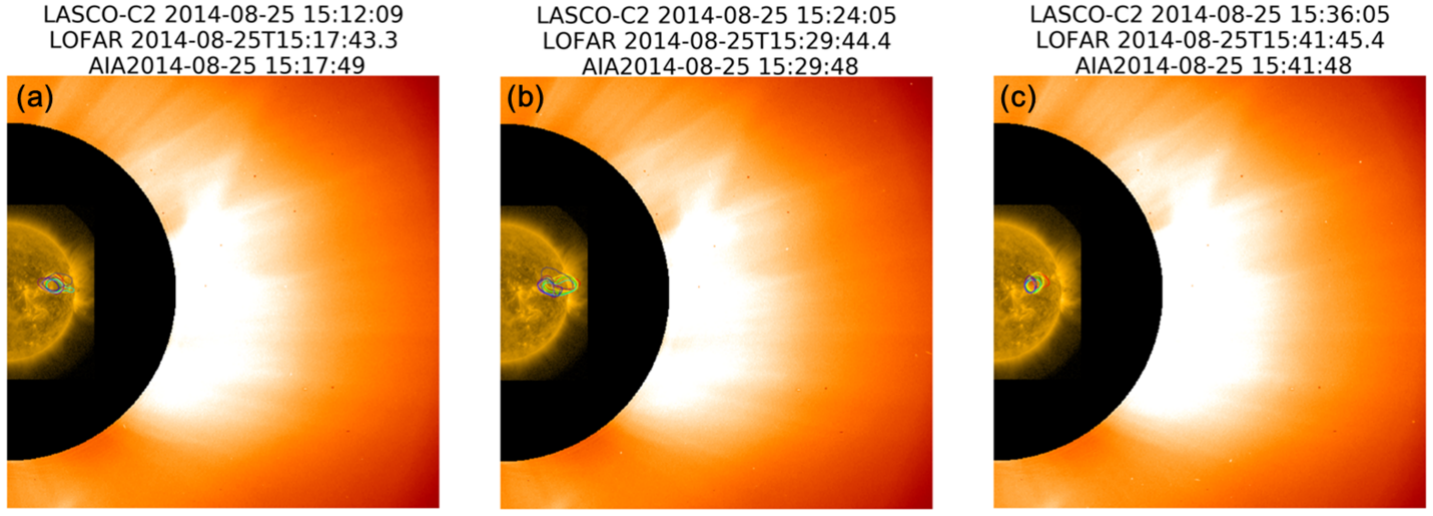
Combined plot of LASCO-C2, LOFAR radio source, and SDO/AIA 171 Å. A CME is visible in LASCO-C2 in the direction of the propagation of the Type-IV.

Figure 6 shows this event in a larger scale, with LOFAR, AIA, and LASCO-C2 on the same panel. We can note the CME eruption in the direction of the propagation of a Type-IV radio source. This CME eruption continued after the Type IV disappeared in the LOFAR HBA frequency range. Unfortunately, LBA radio imaging was not active for this observation and we cannot confirm the association of the lower frequencies radio sources of the Type-IV continuum with the CME core.

### Fine Structures and Emission Mechanism

As already mentioned in Section 1, a Type-IV continuum can have superposed fine structures that have duration much shorter than the Type IV itself. We analyzed the Type-IV fine structure in order to obtain more knowledge on the possible origin. We observe different fine structures, such as spikes, drifting spikes and patchy, and irregular fine structures. To isolate short-duration fine structures, we have plotted the dynamic spectrum in a shorter time range for selected areas within this Type-IV radio burst.

**Figure 7:**
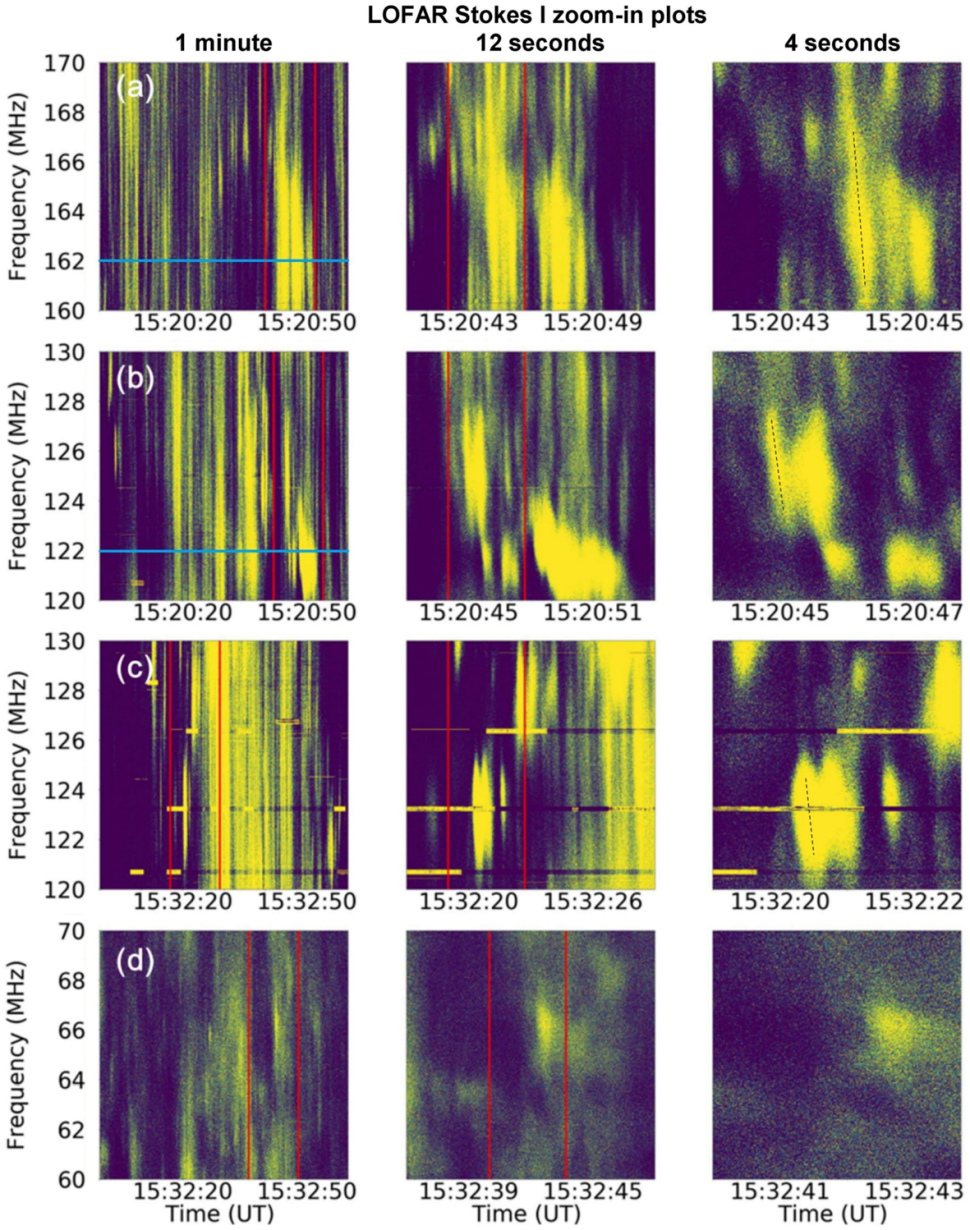
Zoom-in intensity plots of selected areas within the Type-IV radio burst on August 25, 2014. Spectra in the left panels of (**a**), (**b**), (**c**), and (**d**) are 1-minute zoom-in plots of the areas depicted by red box A, B, C, and D in Figure 1; Middle panels of (**a**), (**b**), (**c**), and (**d**) are 12-second zoom-in plots of areas between two red lines in the left panels of (**a**), (**b**), (**c**), and (**d**), respectively; Right panels are 4-second zoom-in plots of areas between the red lines in the middle columns.

Figures 7(a)–(d) are details of the dynamic spectrum within the time and frequency ranges indicated by red boxes A, B, C, and D in Figure 1. Time ranges of left, middle, and right columns are 1 minute, 12 seconds, and 4 seconds, respectively. Despite the short time range, we can see plenty of fine structures that last for about 1 s and less. These narrow-band bursts have the characteristics of spikes, some of them show positive frequency drifts, while others show negative frequency drifts (Bouratzis et al., 2016). We measure the drift rate of the fine structure as denoted with a dotted line in the right panels of Figure 7. We find that the mean drift rate is about 20 MHz/s.

**Figure 8:**
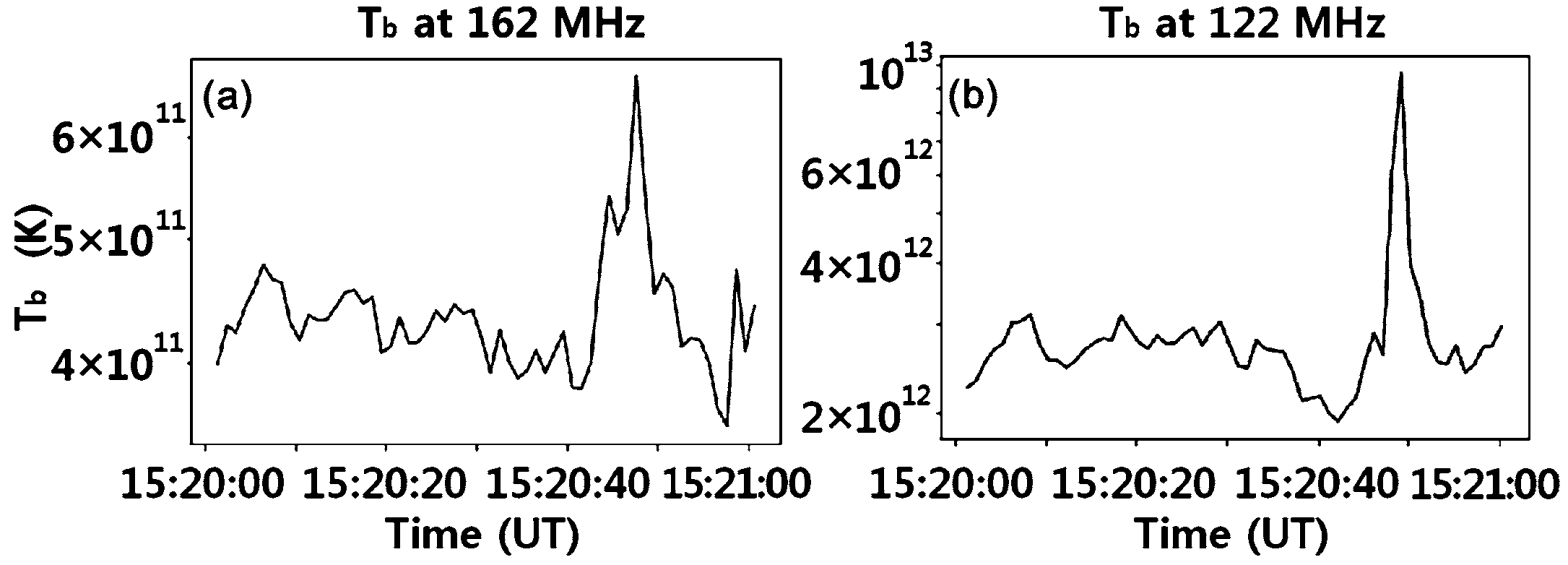
Brightness temperature profiles at 162 MHz and 122 MHz along the blue lines in the left panels of Figures 7(a) and (b), respectively.

Brightness temperature plays an important role in determining the emission mechanism. It can be obtained according to the Rayleigh–Jeans law:

4 $$ T_{\mathrm{B}} = \frac{\lambda ^{2}}{2k\Omega}S , $$

where $T_{\mathrm{B}}$ is the brightness temperature, $\lambda $ is the wavelength, k is the Boltzmann constant, $\Omega $ is the beam solid angle, and S is the flux density. We plot the $T_{\mathrm{B}}$ profiles at 162 MHz and 122 MHz from 15:20 UT to 15:21 UT in Figures 8(a) and (b). $T_{\mathrm{B}}$ data provided by LOFAR have a time resolution of 1 s. Note that these plots are along two blue parallel lines in the left panels of Figures 7(a) and (b). As is shown in Figure 7, there are plenty of fine structures that are brighter than the background Type IV. However, only the most intense fine structures have a brightness temperature noticeably different from the Type-IV continuum. The intense bursts at 162 MHz reach about $6.5\times 10^{11}$ K, while the brightness temperature of the Type-IV background and the weaker fine structures fluctuates around $4\times 10^{11}$ K. The situation is similar at 122 MHz, but with in general higher brightness temperatures. The intense burst has a brightness temperature of about $10^{13}$ K, while background and weaker fine structures have values of about $2.5\times 10^{12}$ K. These examples show that in the case of faint fine structures the brightness temperatures will be of a similar order of magnitude as for the Type-IV continuum.

## Discussion and Conclusions

A moving Type-IV radio burst was observed in the LOFAR dynamic spectrum on August 25, 2014. We performed a detailed comparison of NRH and LOFAR imaging. Having a higher spatial resolution LOFAR observed two separate sources at the onset of the m-Type IV, compared to one unique larger source in NRH. Using the full Stokes parameters from the LOFAR dynamic spectra, we calculated the degree of circular polarization during the propagation of this moving Type IV. The DCP of this Type IV lies in the range 10 – 20% and it is left-hand polarized. The DCP value showed a gradual increase with the development of the Type IV. In addition, the combined LOFAR interferometric data with those of SDO/AIA 171 Å and LASCO-C2 indicate that this Type IV is generated in expanding flare loops located in the CME core. The close-in details of the LOFAR dynamic spectrum showed the existence of countless fine structures. The majority of these look like narrow-band drifting spikes and broadband pulses, and they generally last for less than one second. $T_{\mathrm{B}}$ analysis showed that the most intense fine structures can exhibit a $T_{\mathrm{B}}$ as high as $10^{13}$ K. The Type-IV continuum also showed higher than expected $T_{\mathrm{B}}$ (up to $10^{12}$ K).

We can consider plasma emission, gyrosynchrotron, and ECM as generation mechanisms of the Type-IV background and the fine structures. Our observation presented extremely high brightness temperature and low polarization degree with a gradual increase. Regarding the Type-IV background, the observed gradual rise of DCP value favors gyrosynchrotron radiation as the generation mechanism (Dulk, 1985) because the gradual increase of DCP, as shown in Figure 3, cannot be explained by original plasma emission models (Stewart, 1985).

One possible argument against the gyrosynchrotron emission is that the emission mechanism has difficulty in explaining the high $T_{\mathrm{B}}$. For example, Robinson (1978) found that at polarizations exceeding 40% of the very high brightness temperature (${>}10^{9}$ K) cannot be obtained from gyrosynchrotron emission. We note that DCP is not very high (20 – 30%), and if the local magnetic field allows a very large number of energetic electrons, gyrosynchrotron emission can still be possible. Other physical parameters such as spectral index also need to be inspected in the near future to confirm this.

Fine structures with even higher $T_{\mathrm{B}}$ ($\sim 10^{13}$ K) than the background Type IV may be generated by coherent ECM emission. This speculation needs some justification in the low-frequency regime because the ECM requires a relatively high ambient magnetic field common in the lower corona. Morosan et al. (2016) used a PFSS model and calculated the ECM condition to be ∼ 500 MHz. However, Régnier (2015) used NLFF models and demonstrated that the ECM condition can still be satisfied in the higher corona (1.2 Rs), which supports our conclusion. Another possible interpretation of the results is that a faint gyrosynchrotron background is superposed by high-$T_{\mathrm{B}}$ plasma fine structures. However, we were not able to isolate this faint $T_{\mathrm{B}}$ background in the spectrum due to numerous fine structures superimposed on the continuum.

We conclude that the background Type IV is most likely generated by gyrosynchrotron emission, while the fine structures may still be generated by coherent ECM emission. However, future observations will be necessary to confirm our conclusion on the emission mechanism of moving Type-IV radio bursts and fine structures therein. For example, statistical studies of a number of similar Type-IV bursts will certainly contribute to our understanding of the emission mechanism (Salas-Matamoros and Klein, 2020; Kumari, Morosan, and Kilpua, 2021).

## Supplementary Information

Below are the links to the electronic supplementary material.

(GIF 14.9 MB)

(GIF 19.8 MB)

## Footnotes

1 https://www.solarmonitor.org/index.php?date=20140825&region=12146.

2 https://cdaw.gsfc.nasa.gov/movie/make_javamovie.php?stime=20140825_1401&etime=20140825_1907&img1=lasc2rdf&title=20140825.153605.p270g;V=555km/s.
