# Supplementary figures and images for: Interferometric Imaging, and Beam-Formed Study of a Moving Type-IV Radio Burst with LOFAR
Source: Sol Phys. 2022 Sep 9;297(9):115. doi: 10.1007/s11207-022-02042-0 (PMC9463312; doi:10.1007/s11207-022-02042-0)

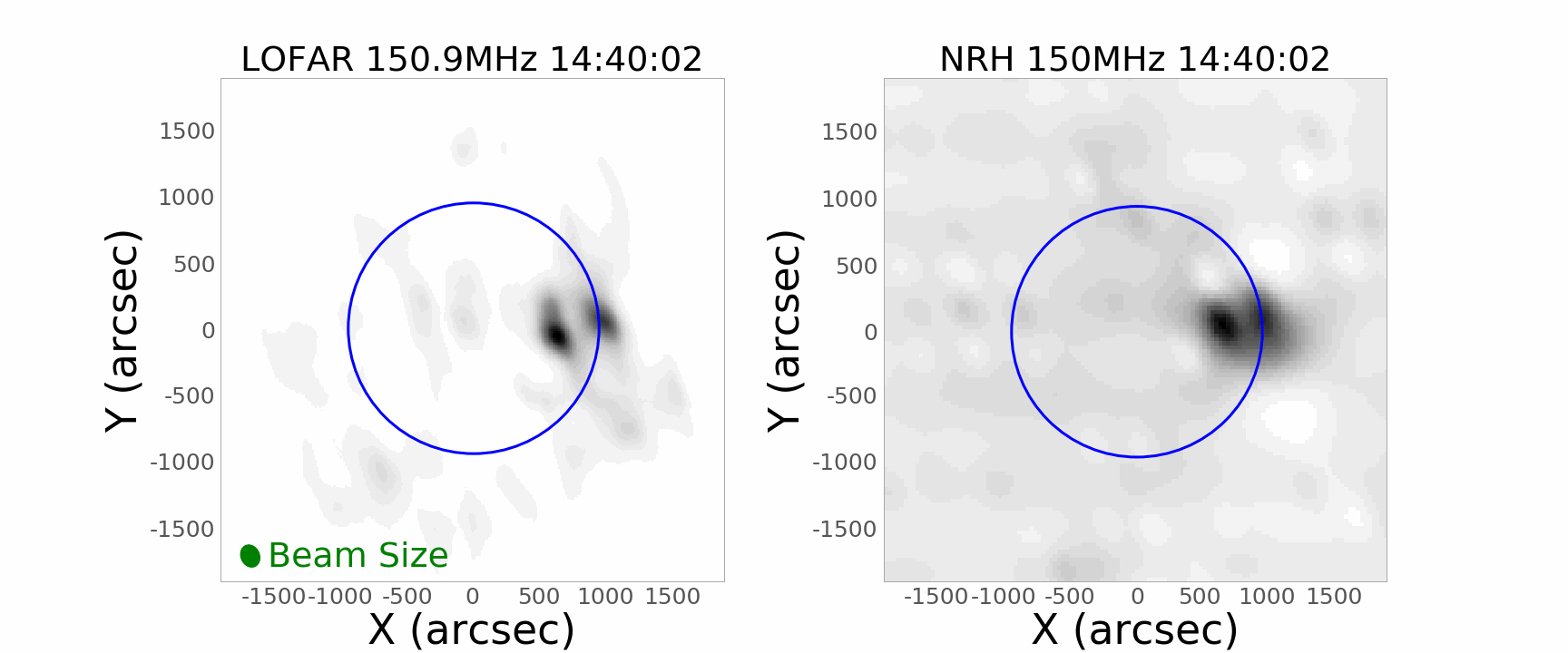

Supplement: Supplementary file 1 — (GIF 14.9 MB) [file 11207_2022_2042_MOESM1_ESM.gif]

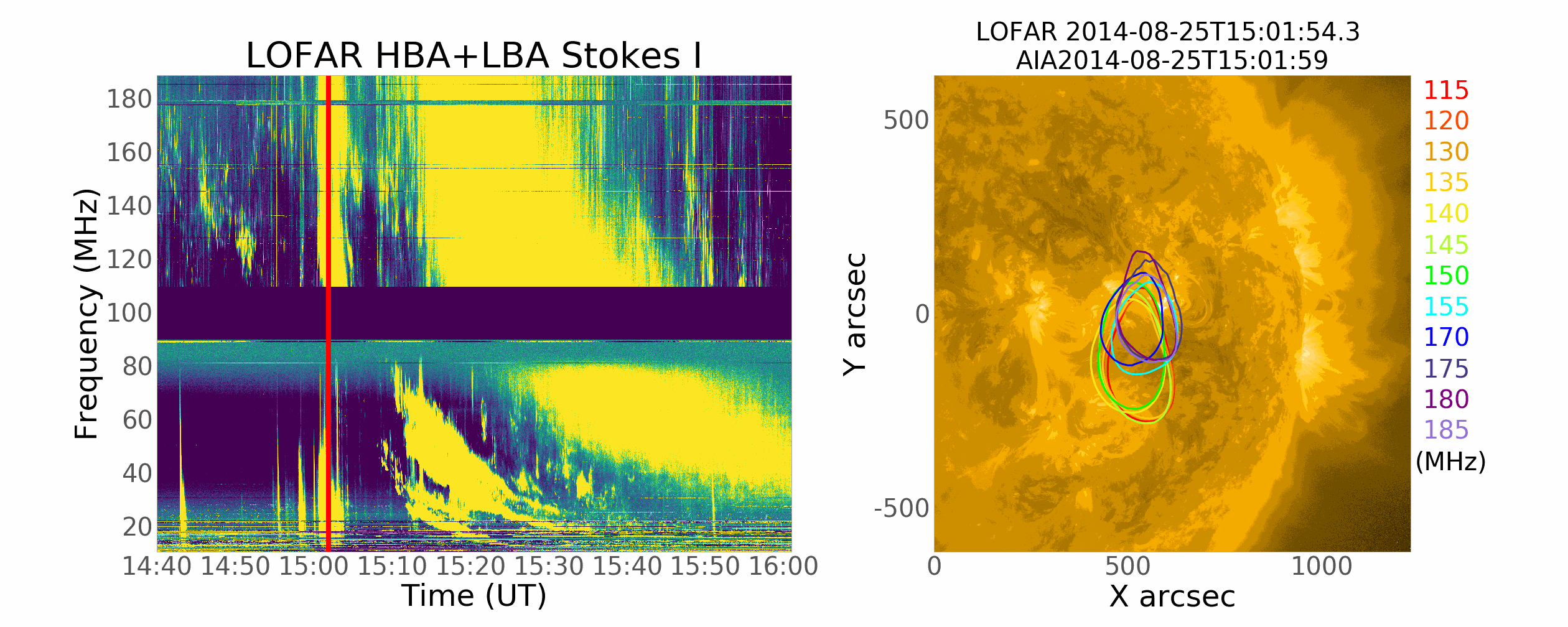

Supplement: Supplementary file 2 — (GIF 19.8 MB) [file 11207_2022_2042_MOESM2_ESM.gif]
